# Aged neuronal nitric oxide knockout mice show preserved olfactory learning in both social recognition and odor-conditioning tasks

**DOI:** 10.3389/fncel.2015.00105

**Published:** 2015-03-27

**Authors:** Bronwen M. James, Qin Li, Lizhu Luo, Keith M. Kendrick

**Affiliations:** ^1^Key Laboratory for NeuroInformation of Ministry of Education, Center for Information in Medicine, University of Electronic Science and Technology of ChinaChengdu, Sichuan, China; ^2^Department of Medicine, St Bernard’s HospitalGibraltar, UK

**Keywords:** aging neuroscience, cognitive decline, nitric oxide, neurodegeneration, neuronal nitric oxide synthase gene, olfactory memory

## Abstract

There is evidence for both neurotoxic and neuroprotective roles of nitric oxide (NO) in the brain and changes in the expression of the neuronal isoform of NO synthase (nNOS) gene occur during aging. The current studies have investigated potential support for either a neurotoxic or neuroprotective role of NO derived from nNOS in the context of aging by comparing olfactory learning and locomotor function in young compared to old nNOS knockout (nNOS^−/−^) and wildtype control mice. Tasks involving social recognition and olfactory conditioning paradigms showed that old nNOS^−/−^ animals had improved retention of learning compared to similar aged wildtype controls. Young nNOS^−/−^ animals showed superior reversal learning to wildtypes in a conditioned learning task, although their performance was weakened with age. Interestingly, whereas young nNOS^−/−^ animals were impaired in long term memory for social odors compared to wildtype controls, in old animals this pattern was reversed, possibly indicating beneficial compensatory changes influencing olfactory memory may occur during aging in nNOS^−/−^ animals. Possibly such compensatory changes may have involved increased NO from other NOS isoforms since the memory deficit in young nNOS^−/−^ animals could be rescued by the NO-donor, molsidomine. Both nNOS^−/−^ and wildtype animals showed an age-associated decline in locomotor activity although young nNOS^−/−^ animals were significantly more active than wildtypes, possibly due to an increased interest in novelty. Overall our findings suggest that lack of NO release via nNOS may protect animals to some extent against age-associated cognitive decline in memory tasks typically involving olfactory and hippocampal regions, but not against declines in reversal learning or locomotor activity.

## Introduction

Many neuronal networks are involved in the decline in cognitive functioning observed during physiological aging and there is evidence that impaired glutamatergic neurotransmission may play a central role. Binding studies have demonstrated an age-related loss of NMDA receptors in different brain regions of rodents and primates (Gonzales et al., 1991; Wenk et al., 1991; Pittaluga et al., 1993). In addition, age-related declines in the amount of mRNA for specific subunits of the NMDA receptor in cortical regions, dentate gyrus and other brain regions have also been reported (Magnusson, 2000; Magnusson et al., 2010). Functional experiments have revealed that the ability of NMDA receptors to stimulate noradrenaline release from hippocampal nerve endings significantly decreases during senescence (Pittaluga et al., 1993) and long-term potentiation (LTP) is attenuated in aged rodents (Barnes and McNaughton, 1985; Deupree et al., 1993).

Along with a role for altered glutamatergic neurotransmission in functional impairments during aging, an involvement of altered nitric oxide (NO) signaling has also been suggested. NO is a diffusible gas molecule in the brain which acts both as a regulator of neurotransmission, synaptic plasticity, neurogenesis, gene expression, neurotoxicity and neuroprotection (Guevara-Guzman et al., 1994; Kendrick et al., 1997; Dawson and Dawson, 1998; Estrada and Murillo-Carretero, 2005). There is much evidence for a dual role of NO in protecting and/or contributing to oxidative damage as a result of aging. Many reports have linked aging with an increase in NO production and its activity as a free radical (McCann et al., 1998; Calabrese et al., 2002; Floyd and Hensley, 2002). While NO is a relatively unreactive radical, it is able to form other reactive intermediates (e.g., NO reacts with the free radical superoxide (O_2_^−^) producing the powerful oxidant peroxynitrite anion (ONOO^−^)). These reactive intermediates can trigger nitrosative damage which in turn may lead to age-related diseases due to the structural alteration of proteins, inhibition of enzymatic activity and interference with regulatory function (Drew and Leeuwenburgh, 2002; Steinert et al., 2010). In support of this, nNOS knockout (nNOS^−/−^) mice are protected from neural damage in a number of models of hypoxia, ischaemia and neurotoxic damage (Morikawa et al., 1992; Kuluz et al., 1993; Ayata et al., 1997; Zaharchuk et al., 1997; Itzhak et al., 1998a,b; Shimizu-Sasamata et al., 1998).

On the other hand, there are several reports suggesting that NO may also possess neuroprotective properties, for example NOS containing neurons are particularly resistant to neurodegeneration in Huntington’s Disease (Ferrante et al., 1985) and excitatory amino acid neurotoxicity (Koh et al., 1996). There is much evidence supporting the cytoprotective actions of NO (Kendrick et al., 1996; Pantazis et al., 1998; Gidday et al., 1999; Teng et al., 1999). However, whether NO is neurotoxic or neuroprotective appears to depend on a number of factors including the concentration of NO (Canals et al., 2001; Calabrese et al., 2007), the isoform involved, the type of cells in which NO is produced and the overall redox state of the environment (Lipton, 1999; Calabrese et al., 2007). Current work is divided over which of the dual roles (neuroprotector/neurotoxic) that NO can play is most important during cerebral aging.

A number of immunocytochemical studies have suggested an important role for NO during aging, however they point to a reduction in NOS containing neurons in several brain regions of the aging rat brain (Cha et al., 1998), particularly the cingulate cortex, parietal area 1, temporal areas 1, 2 and 3, the medial part of occipital cortex area 2, the monocular and binocular part of occipital cortex area 1, the entorhinal cortex, the hippocampus CA1-4, the dentate gyrus and the subiculum. In addition, the number of dendritic branches is decreased (possibly leading to a reduction in released NO) in aged rats and NOS-immunoreactive neurons also tend to become shorter (Cha et al., 1998). On the other hand another study has reported increased brain mitochondrial nNOS expression during aging in rats resulting in a dysfunctional pattern of mitochondrial protein nitration which might contribute to increased apoptosis (Lam et al., 2009). The significance of these age-related changes is not completely established however, although they strongly suggest that alterations in amount and pattern of nNOS expression in the aging brain may be of functional importance.

A reduction in the number of NADPH diaphorase reactive cells (a marker for NOS activity) has been reported in the striatum and olfactory cortex (consisting of the anterior olfactory nucleus and the piriform, the peri-amygdaloid and the entorhinal cortices) in aged rats (Necchi et al., 2002). This reduction is accompanied by a concomitant decrease in the number of nNOS positive cells and the total expression of nNOS protein revealed by immunocytochemistry and Western blotting respectively (Necchi et al., 2002).

Decline in other components of the NO signaling pathway during aging has been investigated using confocal laser scanning microscopy. Circuit-specific alterations of NMDA receptor subunit 1 have been observed in the dentate gyrus of aged monkeys and a reduced number of NADPH diaphorase positive neurones have been detected in the cerebral cortex and striatum (Yamada and Nabeshima, 1998). No age-related changes in NADPH diaphorase activity have been detected in the hippocampus but *in situ* hybridization studies have shown an increase in hippocampal nNOS mRNA expression (Yamada and Nabeshima, 1998).

NO is known to stimulate soluble guanylyl cyclase leading to an elevation of cGMP (cyclic guanosine 3’:5’-cyclic monophosphate). Basal levels of cGMP are maintained by endogenous nitrergic tone (Vallebuona and Raiteri, 1994; Fedele et al., 1996), thus the reduction in activity of nNOS with senescence may contribute to the two-fold reduction in levels of cGMP observed in the hippocampus of rats aged 12 and 24-months old (Vallebuona and Raiteri, 1995). In addition, the activity of soluble guanylyl cyclase (sGC) demonstrates a form of reduced activity in the hippocampus during aging, since hippocampal soluble guanylate cyclase is 30% less responsive to exogenous NO in aged rats when compared to younger controls (Vallebuona and Raiteri, 1995).

The effect of aging and the NOS system has been studied behaviorally using rats in the Morris water maze (Law et al., 2002) where a deficit in spatial memory was observed in some (but not all) rats aged 28-months. In the rats exhibiting the deficit, hippocampal nNOS protein expression was greatly decreased compared to younger rats and the cognitively unimpaired aged rats although their nNOS mRNA expression was increased (Law et al., 2002). It was suggested that the changes in transcriptional activation in older animals might be a compensatory attempt by aged neurones to maintain sufficient neuronal communication and NO balance in the face of a declining NOS-containing neuron population (Law et al., 2002).

A number of studies have used nNOS^−/−^ mice to investigate the role of NO derived specifically from nNOS in terms of neurodegeneration, neuroprotection, neural plasticity and cognitive as well as many other behavioral functions. In the first instance there is strong evidence that nNOS^−/−^ mice, or mice treated with NOS inhibitors, are significantly protected against neurotoxic and ischaemic damage in the brain (Morikawa et al., 1992; Kuluz et al., 1993; Itzhak et al., 1998a,b; Shimizu-Sasamata et al., 1998). Thus it is possible that age-related neurodegenerative changes would be reduced in nNOS^−/−^ leading to reduced cognitive decline. On the other hand a number of experiments in young nNOS^−/−^ mice have found evidence for reduced hippocampal LTP (O’Dell et al., 1994) and for impairments in spatial memory (Kirchner et al., 2004; Tanda et al., 2009; Walton et al., 2013), working memory (Tanda et al., 2009; Zoubovsky et al., 2011) and contextual fear conditioning (Kelley et al., 2009). Thus, it is possible that age-associated cognitive dysfunction in nNOS^−/−^ animals could even be increased compared to control animals, although alternatively reduced neurodegenerative changes might result in a more stable cognitive phenotype during the course of aging.

The current study has therefore investigated the significance of an altered nNOS neuronal signaling system on age-related cognitive decline. There is substantial evidence for the involvement of the NMDA-nNOS-NO-soluble guanylate cyclase signaling cascade in synaptic plasticity associated with olfactory learning (Kendrick et al., 1997; Sanchez-Andrade et al., 2005; Sanchez-Andrade and Kendrick, 2009). NO has also been reported to influence neurogenesis in both olfactory bulb and hippocampus which are important for learning. In mice pharmacological reductions of NO impair both social recognition learning and the social transmission of food preference, although these target NO production from all three NOS isoforms (Sanchez-Andrade et al., 2005). Less is known about the effects of NO derived from nNOS *per se* and particularly in associative learning paradigms involving non-social olfactory cues and the hippocampus (Bunsey and Eichenbaum, 1995). Given evidence in both humans and rodents for age-associated changes in olfactory perception and memory (Doty et al., 1984; Doty, 1989; Mobley et al., 2014) the olfactory system is also a particularly appropriate model for studying interactions between aging and specific genes.

We have therefore utilized olfactory memory and associated reversal learning paradigms in young and old nNOS^−/−^ and wildtype control mice to investigate whether lack of NO derived from nNOS might contribute to age-associated cognitive decline. We chose to use two different olfactory learning tasks; a social recognition memory task (Sánchez-Andrade and Kendrick, 2011) and also a non-social olfactory conditioning task (Brennan et al., 1998). Performance on both of these tasks is impaired by NMDA and AMPA receptor antagonists (James, 2003; Sanchez-Andrade et al., 2005) and involves both the olfactory bulb and hippocampus (Eichenbaum et al., 1989; Pittaluga et al., 1993; Bunsey and Eichenbaum, 1995; Brennan et al., 1998; Sanchez-Andrade et al., 2005; Jüch et al., 2009). There is some evidence that performance on social recognition memory declines with age in rodents (Prediger et al., 2006; Markham and Juraska, 2007), and a previous study has reported only a relatively minor impairment on social recognition memory in young nNOS^−/−^ mice in terms of a reduced memory duration (Jüch et al., 2009). The olfactory conditioning task also allowed us additionally to investigate aging effects on reversal learning. Reversal learning is well established as being sensitive to aging (Schoenbaum et al., 2002; Brushfield et al., 2008) and primarily involves frontal cortical regions (Clark et al., 2004; Mizoguchi et al., 2010). Finally, to investigate more general effects of aging, the locomotor activity of young and old nNOS^−/−^ mice and wildtype controls was assessed using activity boxes.

## Methods

### Animals

The nNOS^−/−^ mice used were a line originally generated by homologous recombination on a mixed B6/129S genetic background and where the targeted deletion of the α-subunit of nNOS resulted in >95% reduction in brain nNOS catalytic activity (Huang et al., 1993). The nNOS^−/−^ animals were originally supplied by Dr Ted Dawson (John’s Hopkins University, Baltimore, USA) and bred in a specific pathogen free barrier unit at the Babraham Institute in Cambridge (UK) together with appropriate genetic background wildtype counterparts (F2 129Sv × C57BL/6J). Four different cohorts of male nNOS^−/−^ and wildtype control animals were used in the experiments: (1) a YOUNG cohort (aged 3–5 months, *n* = 12 wildtype and *n* = 9 nNOS^−/−^); (2) an OLD cohort (aged 18–24 months, *n* = 13 wildtype and *n* = 11, nNOS^−/−^). These first two cohorts were used in both of the olfactory learning tasks and for activity monitoring. Another; and (3) cohort of YOUNG wildtype (*n* = 12) and nNOS^−/−^ (*n* = 12) mice was used in a separate control test for learning speed in the olfactory conditioning paradigm; and (4) a final cohort of YOUNG wildtype (*n* = 24) and nNOS^−/−^ (*n* = 24) mice were used in an experiment investigating effects of the NO-donor molsidomine on the olfactory habituation and social recognition task. Mice were housed in groups of 2–5 under temperature controlled conditions and a 12:12 h light: dark cycle (lights on 07:30). In all cases, housing and rearing conditions were tightly controlled and comparisons between nNOS^−/−^ and wildtype animals were made using age/cohort matched controls. All animals were first raised in the specific pathogen free barrier unit and then moved to a dedicated behavioral testing unit for experimental work. All animals had food (standard REM rodent diet) and water available *ad libitum* (other than during a period of restricted feeding prior to the olfactory conditioning task). They were handled daily for at least 5 days before behavioral tests were carried out and at least weekly for the rest of the time. The breeding of nNOS^−/−^ animals and all experiments were carried out in strict accordance with UK Home Office guidelines and specifically licensed under the Animals (Scientific Procedures) Act 1986.

### Genotyping Using PCR

Tail biopsies were taken from all experimental animals post-mortem. The tail tissues were placed in 500 µL of tail lysis buffer (100 mM EDTA, pH 8.0, 50 mM Tris-HCL pH 8.0, 0.5% SDS (w/v), 50 µg/ml proteinase K (Promega)) and digestions were then incubated at 55°C for 12-h. Genotyping confirmed that the nNOS/neomycin junction was present in all nNOS^−/−^ mice and absent in all wildtype mice. Genotyping using PCR required two independent reactions. The wildtype allele was identified with a forward primer: that annealed to part of the nNOS gene; and a reverse primer that annealed to nNOS itself, (the primer pair generated a 500 bp PCR product):

- 5′- ATCTCAGATCTGATCCGAGGAG-3′
- 5′- CTTTCATCTCTGCTTTGGCTGG-3′

The mutant allele was detected using a separate primer pair: a forward and reverse primer both directed to the nNOS/Neomycin junction and which generated a 700 bp PCR product:

- 5′- TCGCCTTAACGCGGTGCCCTG-3′
- 5′- CTCCCGATTCGCAGCGCATCG-3′

To amplify sequences relating to each genotype the same reaction conditions were set up in Stratagene thin walled tube strips. Separate were run for each primer set thus there were two separate reactions for each DNA sample. The DNA samples from the tails samples were re-suspended in 100 µl of TE buffer (10 mM Tris (pH 8.0) and 1.0 mM ethylenediamine tetra-acetic acid). Once the PCR reaction was complete a 1.5% agarose gel was made by dissolving agarose (iGI or GIBCO BRL) in 1 × buffer containing 45 mM Tris/Borate, 2 M EDTA pH 8.0 and heating in a microwave until molten. Ethidium Bromide was added to a final concentration of 0.5 µg/ml mixed by swirling and left to cool. Gels were poured when the temperature of the molten agarose had fallen below 60°C and left to solidify. Gels were submerged in 1 × TBE buffer and the samples were loaded along with 10 µl dye per well and a DNA ladder and then were run at 100 V/cm. The nucleic acids were then visualized under UV illumination.

### Olfactory Habituation and Social Recognition Memory

In this task YOUNG and OLD nNOS^−/−^ and wildtype animals were repeatedly exposed to the same adult stimulus animal (male C57/Bl6 × 129sv) to establish if they showed a progressive reduction in investigation time indicative of their both being able to detect its odor and habituate to repeated exposure to it. The adult male stimulus animals were anesthetized (Hypnorm/Hypnovel 6 mg/kg i.p.) so as to remove any influence of their behavior on the test animals. This was of particular importance in the context of the current experiments in view of previous reports of altered aggression (Nelson et al., 1995), social motivation and stress behaviors in nNOS^−/−^ animals (Tanda et al., 2009; Walton et al., 2013). Animals were then returned to their home cages overnight before being tested for their ability to remember the same stimulus mouse compared with a novel one 24 h later, as indexed by a reduced investigation time of the familiar compared with the unfamiliar stimulus animal.

In this paradigm experimental animals were first habituated to a testing arena (A Perspex round bottomed bowl, Bioanalytical Systems, USA: diameter = 90 cm at top and 45 cm at base and height = 60 cm) for 10-min before being presented with a sedated adult stimulus mouse for 1-min. After a further 10-min, the test mice were presented with the same stimulus mouse for another 1-min trial. The same procedure occurred for two more trials making a total of four trials with the same stimulus animal. Ten minutes after the fourth trial the test animal was returned to its home cage overnight. In between trials the sedated stimulus mouse was replaced into a single cage on a warming plate. At the end of the day, and after a full recovery, the sedated mouse was returned to its home cage.

The following day the test mouse was habituated to the arena for 10 min before being presented with two sedated stimulus mice for a test time of 2-min: one being the familiar mouse presented the previous day on trials 1–4 and the other a novel mouse (not used as a stimulus mouse in any previous trial). The time spent investigating each mouse was recorded using a handheld stopwatch. The behaviors were all videotaped to allow blind scoring of the investigation times.

A further experiment using this paradigm was carried out on the fourth cohort of young (3–5 months old) nNOS^−/−^ (*n* = 24) and wildtype (*n* = 24) animals. Here half the animals in each group (i.e., *n* = 12) were either given the NO donor molsidomine (10 mg/kg, i.p. Sigma UK, dissolved in saline) or saline (1 ml/kg, i.p.) as a control. Molsidomine is a prodrug, and upon metabolism releases NO and an active metabolite (3-morpholinosydonimine, SIN-1), used clinically in the treatment of angina pectoris (Rosenkranz et al., 1996). Animals were injected 30-min prior to the first exposure to the stimulus mouse in the habituation trials on day 1. The timing and dose of molsidomine given were based on results from several previous studies demonstrating its ability to reverse effects of NOS inhibitors in object recognition memory tasks (Meyer et al., 1998; Pitsikas et al., 2002, 2003a). All mice were given saline injections (1 ml/kg, i.p.) for 3 days prior to commencement of testing in order to habituate them to any effects of injection stress.

### Olfactory Conditioning Paradigm

An olfactory conditioning procedure was used to promote recognition memory for artificial odors. For this, lemon or peppermint food essence was combined with a sugar reward similar to the paradigm reported by Brennan et al. (1998). Mice in both the YOUNG and OLD groups were initially group housed and handled daily. They were then singly housed for 4 days in a procedure room on a 12 h light/dark cycle (lights on at 07:00) with free access to food and water. Animals were handled and weighed each day so that a stable baseline weight could be calculated. They were then placed on a restricted feeding regime in which 2 g of standard rodent chow was given each day at 17:00 in order to maintain their body weight at 85% free-feeding weight. The training began 3 days after the initiation of the food restriction.

Half the mice in each group were trained to associate the sugar reward with lemon odor (conditioned odor, CS+; SuperCook, Sherburn-in-Elmet, Leeds, U.K.). Peppermint odor (SuperCook, Sherburn-in-Elmet, Leeds, U.K.) served as the non-conditioned stimulus (CS−) presented in the absence of sugar. For the other half of the mice, peppermint was presented as the CS+ and lemon as the CS−. The odors were presented by sprinkling 50 µl of the food essence over clean sawdust in a plastic petri dish (Sterilin, 10 cm diameter). In the dish containing the conditioned odor, small fragments of sugar were placed beneath the sawdust during training.

The conditioning trials took place in cages identical to the home cage but with no sawdust/food/water. A trial consisted of placement of a dish containing either CS+ or CS− in the cage for a period of 10-min. At the end of each trial, the mice were returned to their home cages and the dishes washed thoroughly in hot water and Hibiscrub/70% alcohol. All mice received two training sessions per day over 2–3 days with two CS+ and two CS− trials per session (i.e., 16–24 trials in total). Individual training sessions were given 2 h apart and the order of the trials was chosen pseudo-randomly over the eight daily trials with a maximum of three CS+ or CS− trials permitted in a row.

To determine whether the mice had learned to differentiate between the CS+ and CS− odors a preference test was conducted on the day following the final conditioning session using two black Perspex compartments (height: 30 cm × width: 30 cm × depth: 30 cm) linked by a connecting passage (height: 30 cm × width: 10 cm × depth: 10 cm). Dishes containing sawdust were placed in each chamber. Prior to preference testing the mice were habituated to the apparatus for 5 min by placing them in the central connecting passage and allowing them to investigate freely. The dishes were then removed briefly in order to add 50 µl of lemon odor to one dish and 50 µl peppermint to the other in the absence of sugar. The mouse was replaced in the central section and its behavior recorded for 5-min by an observer using a hand held stopwatch. The total time spent in each compartment and the amount of time digging in each dish was recorded. In order to remove any odor trails the apparatus was thoroughly cleaned and wiped dry using 2% acetic acid between training sessions and between testing.

The paradigm was extended further to establish an index of reversal learning; such that the following day mice were re-trained using reversed contingencies (i.e., 1 day of reversal training with 4 trials, 2 × new CS+ and 2 × new CS−) and were then re-tested the subsequent day. Using this approach we could establish whether animals persevered with their original learning (i.e., continued to show a significant preference for the original CS+ odor), or showed extinction of the original learning indicating progress towards a reversal (i.e., showed no significant preference for the new CS+ compared to the new CS− odor), or showed a complete reversal (i.e., showed a significant preference for the new CS+ odor).

### Locomotor Activity Tests

Locomotor activity was assessed using a battery of clear Perspex photo-beam boxes (width = 21 cm × height = 20 cm × depth = 36 cm; Tech^nix^, Babraham Institute, U.K). These contained two transverse infrared beams 10 mm from the base spaced equally along the length of the box. A computer recorded beam breaks and runs (where the front and rear beams are broken in close succession) in 5 min time bins. Animals were placed in the boxes (which were cleaned between animals using 2% acetic acid) for 2 h on three consecutive days, always at the same time of day (each testing session began at the same time each day between 08:00 and 16:00). This design allowed assessments of novelty reactivity (including sensitivity and habituation) as well as basic locomotor competence.

### Statistics

For the olfactory habituation and social recognition task investigation times of the four successive habituation trials were compared using a 2-way repeated measures ANOVA, with factors (GENOTYPE (wildtype and nNOS^−/−^) and TRIAL (1–4)). The investigation times for the 24 h memory test (familiar compared with unfamiliar) were compared within groups using a paired *t*-test. For the experiment investigating effects of molsidomine in YOUNG animals a 2-way ANOVA was carried out with factors (GENOTYPE (wildtype or nNOS^−/−^ and TREATMENT (molsidomine or saline)) for the habituation phase and factors (TREATMENT and FAMILIARITY (familiar and unfamiliar stimulus animals)) for the 24 h social recognition memory phase. For the olfactory conditioning experiment compartment preference and digging time data were subjected to 2-way ANOVAs with factors (GENOTYPE × ODOR (CS+ or CS−)). In order to compare the effect of age and genotype directly a preference index for both the compartment and digging preference data was calculated: (Compartment preference index = time spent in the compartment containing the CS+ minus time spent in compartment containing CS−)/(time spent in CS+ compartment plus time spent in CS− compartment; Digging preference index = calculated the same way, but using time spent digging in CS+ and CS− odor dishes). To investigate aging effects a 2-way ANOVA was then performed with factors AGE and GENOTYPE. In order to analyze the reversal data the preference indices were compared using an unpaired *t*-test. For the locomotor activity experiments data was collected and analyzed by comparing the total number of beam breaks and runs between groups using a 2-way ANOVA with factors (TIME (5 min time bins) and SESSION (Days 1–3)). To test for age differences a 2-way ANOVA with factors (GENOTYPE × AGE) and *post hoc* Tukey test was performed using mean activity and beam break data.

## Results

### Olfactory Habituation and Social Recognition Memory in Young and Old nNOS^−/−^ and Wildtype Animals

**Figure 1:**
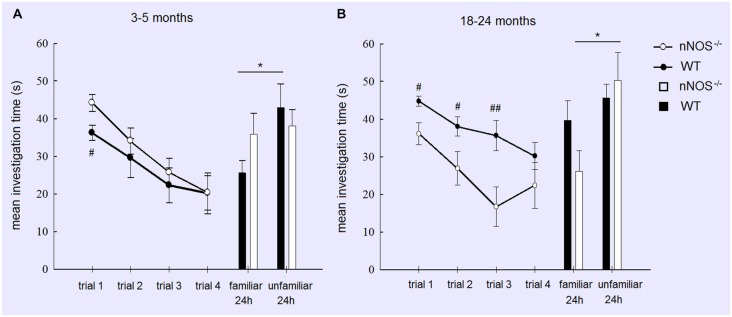
**Performance of YOUNG (A) and OLD (B) nNOS and wildtype mice in the social recognition task**. Graphs show mean ± sem habituation of olfactory investigation times across 4 × 1 min trials separated by 10 min where test animals are exposed to an anesthetized adult stimulus. Histograms show mean ± sem investigation times of a familiar vs. unfamiliar stimulus mouse (presented together) in a 2 min test given 24 h after the habituation trials. Both YOUNG and OLD mice from the two groups show significant habituation of investigation across the 4 trials (see text) but only YOUNG wildtype and OLD nNOS^−/−^ animals show significantly greater investigation time for the unfamiliar vs. familiar stimulus mice indicative of the formation of a long term recognition memory. *P < 0.05 two-tailed *t*-test unfamiliar vs. familiar; ##*P* < 0.01, #*P* < 0.05 two-tailed *t*-tests wildtype vs. nNOS^−/−^.

For the YOUNG groups a two way ANOVA with GENOTYPE and TRIAL as factors revealed no significant effect of GENOTYPE (*F*_1,80_ = 1.97, *P* = 0.164) and a significant effect of TRIAL (*F*_3,80_ = 9.97, *P* < 0.001), but no GENOTYPE × TRIAL interaction (*P* > 0.1). This indicates that overall both nNOS and wildtype animals displayed equivalent amounts of investigation and a similar reduction due to habituation across trials. However, an exploratory analysis using a *t*-test did show that nNOS^−/−^ mice spent significantly more time investigating the stimulus animal on Trial 1 (*P* < 0.05—see Figure 1). YOUNG nNOS^−/−^ mice were impaired in their ability to discriminate between the familiar and unfamiliar stimulus mice at the 24 h test (*t*_11_ = 0.2876, *P* = 0.7790) whereas the wildtype controls showed a clear discrimination spending significantly more time investigating the unfamiliar stimulus animal (*t*_9_ = 2.689, *P* = 0.0248).

A similar analysis of the OLD groups also revealed a significant main effect of TRIAL (*F*_3,76_ = 5.92; *P* = 0.001) indicating that both OLD nNOS^−/−^ and wildtype groups of mice habituated similarly to the presence of the stimulus mouse by showing reduced investigation times over trials. This suggests that their ability to detect olfactory cues from the stimulus animal was unimpaired. However, there was also a main effect of GENOTYPE (*F*_1,76_ = 18.15, *P* < 0.001) due to wildtype control mice spending significantly longer investigating the stimulus animal. While there was no GENOTYPE × TRIAL interaction (*F*_3,76_ = 0.85, *P* = 0.471), exploratory *t*-tests revealed significant differences between investigation times in the two groups in trials 1, 2 and 3 (see Figure 1). While OLD wildtype mice did not show a significant discrimination between familiar and unfamiliar stimulus mice at the 24 h test (*P* > 0.1), nNOS^−/−^ mice did (*P* < 0.05—see Figure 1).

### Effects of Molsidomine Treatment on Olfactory Habituation and Social Recognition Memory in Young Animals

**Figure 2:**
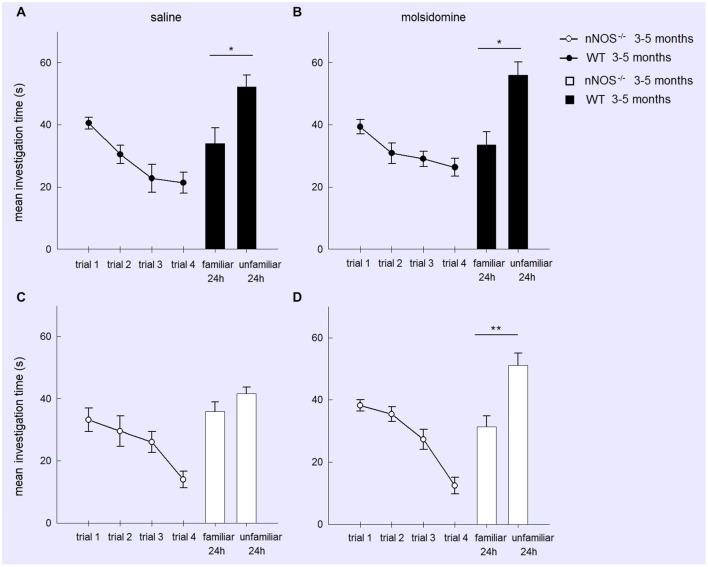
**Treatment with the NO donor molsidomine rescues the olfactory memory deficit in young nNOS^−/−^ mice in the social recognition task**. Graphs show mean ± sem habituation of olfactory investigation times across 4 × 1 min trials separated by 10 min where test animals are exposed to an anesthetized adult stimulus in the four treatment groups (Wildtype **(A)** or nNOS^−/−^ **(C)** saline control; Wildtype **(B)** or nNOS^−/−^ **(D)** molsidomine treated). There were no significant differences in investigation times or habituation between the groups. Histograms show mean ± sem investigation times of a familiar vs. unfamiliar stimulus mouse (presented together) in a 2 min test given 24 h after the habituation trials. nNOS^−/−^ animals treated with saline do not show a significant difference in investigation times whereas nNOS^−/−^ animals treated with molsidomine do. Saline and molsidomine treated wildtype animals also show a similar significant difference. *P < 0.05, **P < 0.01 two-tailed unfamiliar vs. familiar stimulus animal (*t*-test).

In order to test whether memory deficits in YOUNG nNOS animals in the social recognition task were due to a lack of NO produced by other NOS isoforms we performed an additional experiment where we tried to rescue their deficit by treatment with the NO-donor molsidomine. A two-factor ANOVA showed there was no effect of molsidomine on the behavior of the YOUNG wildtype or nNOS^−/−^ mice during the habituation phase (TREATMENT: *F*_1,188_ = 1.97, *P* = 0.163; GENOTYPE: *F*_1,188_ = 2.63, *P* = 0.106; TREATMENT × GENOTYPE interaction: (*F*_1,188_ = 0.004, *P* = 0.991). Molsidomine did not appear to influence the presence of a normal recognition memory at 24-h (evidenced by increased investigation time of the novel stimulus animal) in wildtype control mice (saline treated: *t*_11_ = 2.352, *P* = 0.0384; molsidomine treated: *t*_11_ = 2.827, *P* = 0.0165). However, while in saline treated nNOS^−/−^ mice there was no evidence for recognition memory at 24 h (*t*_11_ = 1.45, *P* = 0.1739) in molsidomine treated animals there was (*t*_11_ = 3.388, *P* = 0.0061) (Figure 2).

A further analysis of treatment effects in the two genotypes (wildtype and nNOS^−/−^) was performed using 2-way ANOVAs with factors FAMILIARITY (i.e., unfamiliar and familiar stimulus animals) and TREATMENT. In wildtype animals there was a significant effect of FAMILIARITY (*F*_1,44_ = 21.60, *P* < 0.001) but no effect of TREATMENT (*F*_1,44_ = 0.15, *P* = 0.697) or FAMILIARITY × TREATMENT interaction (*F*_1,44_ = 0.22, *P* = 0.642). Thus saline and molsidomine treated animals showed a similar degree of preference for investigating the novel stimulus animals. In nNOS^−/−^ mice on the other hand while there were no main effects of FAMILIARITY or TREATMENT there was a significant FAMILIARITY × TREATMENT interaction (*F*_1,44_ = 4.77, *P* = 0.034) showing that molsidomine treatment produced a significant change compared to saline and rescued the recognition memory deficit.

### Olfactory Conditioning and Reversal Learning in Young and Old nNOS^−/−^ and Wildtype Animals

**Figure 3:**
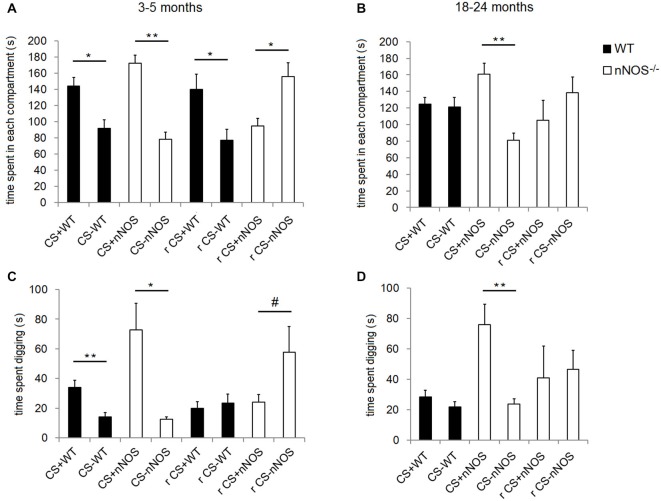
**Performance of YOUNG and OLD nNOS and wildtype mice in the olfactory conditioning task**. Histograms show mean ± sem time spent in the compartments with the CS+ and CS− odors (preference behavior) in YOUNG **(A)** and OLD **(B)** wildtype and nNOS^−/−^ mice and also the amount of time spent digging in the shavings with the CS+ and CS− odors **(C)** and **(D)**. Data is given both for the original CS+ and CS− contingency and also following reversal learning (r). There is no reversal learning data for old wildtype animals since they did not learn the original contingency. *P < 0.05, **P < 0.01, #*P* < 0.05 one-tailed CS+ vs. CS− (*t*-test).

Analyses of the data within YOUNG groups of animals revealed that both nNOS^−/−^ and wildtype control mice spent significantly longer in the compartment containing the CS+ odor (nNOS^−/−^: *t*_10_ = 4.844, *P* = 0.0007; wildtype: *t*_12_ = 2.589, *P* = 0.0237—see Figure 3). This result was paralleled in the digging preference data which revealed that both groups spent significantly longer digging in the dish containing the CS+ odor (nNOS^−/−^: *t*_7_ = 3.441, *P* = 0.0108; wildtype: *t*_8_ = 4.477, *P* = 0.0021). When a 2-way ANOVA was performed with factors GENOTYPE and ODOR, both nNOS^−/−^ and wildtype controls showed a significant preference for the compartment containing the conditioned odor (ODOR: *F*_1,44_ = 47.71, *P* < 0.001), and no differences were observed between groups in the amount of time spent in each compartment (GROUP: *F*_1,44_ = 0.45, *P* = 0.501). There was a marginal GENOTYPE × ODOR interaction (*F*_1,44_ = 3.95, *P* = 0.053). The digging preference data revealed that both groups spent significantly more time digging in the dish containing the CS+ odor (ODOR: *F*_1,30_ = 18.86, *P* < 0.001). However, a significant effect of GENOTYPE was also observed (*F*_1,30_ = 4.31, *P* = 0.047) and a GENOTYPE × ODOR interaction (*F*_1,30_ = 5.12, *P* = 0.031) showing that the nNOS^−/−^ mice spent significantly more time digging in the dish containing the CS+ odor.

In order to control for the possibility that the YOUNG nNOS^−/−^ mice might be learning the conditioning task more quickly than wildtype controls (resulting in an apparent advantage in reversal learning after 1 day of training), two other cohorts of YOUNG mice naïve to the test (*n* = 12 nNOS^−/−^ and *n* = 12 wildtype) were trained on the original task for only 1 day (i.e., 4 trials) and then tested on the subsequent day. Neither the wildtype controls nor the nNOS^−/−^ mice showed a preference for the compartment containing the conditioned odor after a single day of training (wildtype: *t*_11_ = 0.3530, *P* = 0.7307 and nNOS^−/−^: *t*_11_ = 1.384, *P* = 0.1938) and further analyses using a 2-way ANOVA revealed no significant effect of GENOTYPE (*F*_1,44_ = 0.21, *P* = 0.648) or ODOR (*F*_1,44_ = 2.65, *P* = 0.111). There was also no GENOTYPE × ODOR interaction (*F*_1,44_ = 0.77, *P* = 0.386). However, the digging preference data did reveal a significant effect of ODOR (*F*_1,42_ = 15.38, *P* < 0.001), although both wildtype and nNOS^−/−^ mice showed a significant preference for the conditioned odor. There was no significant effect of GENOTYPE (*F*_1,42_ = 0.62, *P* = 0.434) or GENOTYPE × ODOR interaction (*F*_1,42_ = 0.19, *P* = 0.666). This control experiment therefore provided no evidence that YOUNG nNOS^−/−^ mice could learn the task better than wildtypes after 1 day of training and so the advantages we found for nNOS^−/−^ animals in reversal learning are unlikely to have been due to a superior learning speed to that in wildtype controls.

Analyses of the compartment preference data using a 2-way ANOVA in the OLD animals revealed no significant effect of GENOTYPE on the amount of time spent in each compartment (*F*_1,38_ = 0.03, *P* = 0.861) but a significant effect of ODOR (*F*_1,38_ = 11.68, *P* = 0.002). There was also a significant GENOTYPE × ODOR interaction (*F*_1,38_ = 12.88, *P* < 0.001) confirming that the nNOS^−/−^ mice demonstrated a significant preference for the CS+ containing compartment whereas the wildtype controls did not. Analyses of the digging preference data revealed significant effects of GENOTYPE (*F*_1,38_ = 13.19, *P* < 0.001) and ODOR (*F*_1,38_ = 15.07, *P* < 0.001) and a GENOTYPE × ODOR interaction (*F*_1,38_ = 11.53, *P* = 0.002), which supported the compartment preference finding (see Figure 3).

In order to test for an interaction with age and genotype a 2-way ANOVA was performed with factors AGE and GENOTYPE using the preference indices. For the compartment preference data the main effect of AGE was marginally significant (*F*_1,41_ = 3.28, *P* = 0.078) and a significant main effect of GENOTYPE was observed (*F*_1,41_ = 7.50, *P* = 0.009). The lack of an age effect could be related to the fact that the mice tended to sit in the connecting channel between the two compartments. No AGE × GENOTYPE interaction was found (*F*_1,41_ = 0.90, *P* = 0.349). When the digging preference data were analyzed a significant effect of both AGE (*F*_1,34_ = 6.23, *P* = 0.018) and GENOTYPE (*F*_1,34_ = 7.17, *P* = 0.011) were observed but there was no AGE × GENOTYPE interaction (*F*_1,34_ = 0.11, *P* = 0.745).

When the contingencies were reversed, (i.e., mice previously conditioned to associate lemon odor with a sugar reward were then trained to associate peppermint with the sugar reward or *vice versa)*, the YOUNG wildtype mice spent significantly more time in the compartment containing the odor to which they were conditioned originally (*t*_12_ = 2.540, *P* = 0.0260); i.e., they persevered with the original learning. However, the YOUNG nNOS^−/−^ mice showed a clear preference for the compartment containing the odor to which they had been most recently trained (*t*_9_ = 2.674, *P* = 0.0254; i.e., they showed reversal of original learning). The digging preference data revealed that nNOS^−/−^ mice appeared to spend more time digging in the dish containing the new conditioned odor, however this did not quite achieve significance (*t*_9_ = 1.847, *P* = 0.0979). The wildtype control mice spent approximately equal time digging in each dish (*t*_11_ = 0.7259, *P* = 0.4831—see Figure 3).

When the OLD nNOS^−/−^ mice were tested for learning a reversal of task contingencies, in compartment preference or digging preference indices (*t*_8_ = 0.8695, *P* = 0.4099 and *t*_7_ = 0.2128, *P* = 0.8375). However, analysis of the side preference index data for reversal learning using an unpaired *t*-test revealed a trend towards a difference between the YOUNG and OLD nNOS^−/−^ mice (*t*_16_ = 1.869, *P* = 0.0800). There was a similar non-significant trend for digging preference (*t*_15_ = 1.727, *P* = 0.1048—see Figure 3).

### Locomotor Activity in Young and Old nNOS^−/−^ and Wildtype Animals

**Figure 4:**
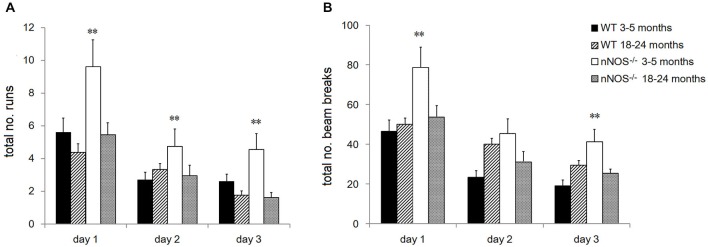
**Performance of YOUNG and OLD nNOS and wildtype mice in the locomotor activity task**. Histograms show mean ± sem total number of **(A)** runs and **(B)** beam breaks by individual animals in YOUNG and OLD wildtype and nNOS^−/−^ animals. **P < 0.01 two-tailed Tukey test vs. all other groups.

**Figure 5:**
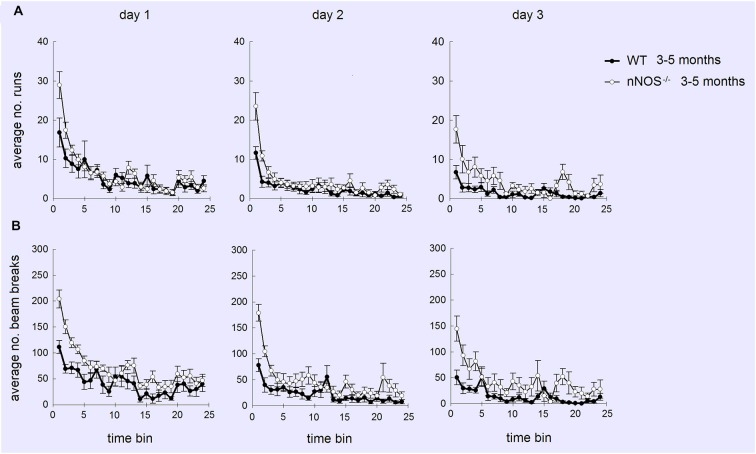
**Profiles of numbers of runs (A) and beam breaks (B) displayed by YOUNG wildtype and nNOS^−/−^ mice in activity boxes for 25, 5 min time bins (2 h)**. Activity was monitored over 3 successive days. It can be seen that increased activity (mean ± sem) in nNOS^−/−^ animals is greatest on day 1 and occurs mainly during the first 25–30 min (5–6 time bins) of the tests.

YOUNG nNOS^−/−^ mice were significantly more active than their wildtype controls over the three activity sessions in terms of the total number of runs and number of beam breaks in each 2 h activity session (see Figure 4). Further analyses of the 5 min time bin data using a 2-way ANOVA revealed significant main effects of GENOTYPE and TIME BIN for both the run and the beam break data over the 3 days (day one run data: effect of GENOTYPE *F*_1,456_ = 35.47, *P* < 0.001, and TIME BIN *F*_23,456_ = 20.51, *P* < 0.001). The increased activity levels were apparent during the first 25–30 min of each test session, after which there appeared to be no difference between groups (see Figure 5). There was a decrease in activity, common to both groups, that occurred from test day one to test day three (effect of SESSION on total number of runs: *F*_2,1338_ = 40.79, *P* < 0.001).

No significant differences were observed in the activity of the OLD nNOS^−/−^ and the wildtype control mice aged 18–24 months over the 3 days of testing, when the overall number of runs and beam breaks in each session were compared between groups (see Figure 4). There was however a decrease in activity, common to both groups that occurred from test day one to test day three (effect of SESSION on total number of runs: *F*_2,1722_ = 49.12, *P* < 0.001).

Detailed analyses of the activity data using a two-way ANOVA with factors AGE and GENOTYPE revealed a significant effect of age on day one with the older mice making fewer numbers of runs and beam breaks in each session than their younger counterparts (*F*_1,92_ = 6.71; *P* = 0.011—see Figure 4). This was not evident on days two and three. In addition, there was a significant effect of GENOTYPE with the nNOS^−/−^ mice making more runs and beam breaks in each session compared to their wildtype controls (*F*_1,92_ = 5.97, *P* = 0.016). However, there was no significant interaction between AGE and GENOTYPE (*F*_1,92_ = 1.94; *P* = 0.167) indicating that the decline in activity observed was not exclusive to either the nNOS^−/−^ or wildtype mice.

## Discussion

The current studies using nNOS^−/−^ mice have provided support for the hypothesis that NO derived from nNOS may contribute to age-associated neurodegenerative changes that lead to cognitive decline using two different olfactory learning paradigms. However, there appeared to be no evidence for a similar protection against age-associated decline in reversal learning or locomotor activity. Tasks involving social recognition and olfactory conditioning paradigms showed that old aged (18–24 months) nNOS^−/−^ animals showed improved retention of learning compared to similar aged wildtype controls. Young (3–5 months) nNOS^−/−^ animals showed superior reversal learning to wildtypes in the conditioned learning task, the later showing perseveration of learning (i.e., they failed to learn the reversal contingency). On the other hand old aged nNOS^−/−^ animals failed to show complete reversal learning during the same 1-day time-period. Whereas young nNOS animals were impaired in long term memory for social odors compared to wildtype controls, in old aged animals this pattern was reversed, possibly indicating beneficial compensatory changes influencing olfactory memory may occur during aging in nNOS animals. Both nNOS^−/−^ and wildtype animals showed an age-associated decline in locomotor activity although young nNOS^−/−^ animals were significantly more active than wildtypes. Overall our findings suggest that lack of NO release via nNOS may protect animals to some extent against age-associated cognitive decline in memory tasks typically involving olfactory and hippocampal regions, but not against declines in reversal learning or locomotor activity.

The current study provided evidence for an olfactory social recognition memory impairment in young nNOS^−/−^ animals since they failed to show significantly differential investigation times between familiar and unfamiliar stimulus animals after 24 h. This is in agreement with a previous study using a juvenile recognition version of this paradigm and which reported a similar memory impairments in nNOS^−/−^ animals at 24 h although not after 6 h or less (Jüch et al., 2009). However, while old wildtype control animals lost their ability to exhibit a recognition memory after 24 h, old nNOS^−/−^ animals actually gained this ability, having been unable to do this at a younger age. Thus in this context not only do nNOS^−/−^ animals appear to be protected against age-associated cognitive decline but actually displayed a gain of function suggestive of positive compensatory changes over the course of aging.

Another recent study has reported that nNOS^−/−^ animals do not show a preference for social novelty in a three chamber preference test where time spent with a socially familiar or unfamiliar stimulus animal is recorded (Walton et al., 2013). It is therefore possible that this might have contributed to the absence of recognition memory we found in young nNOS^−/−^ animals. However, Jüch et al. (2009) reported a preference for a novel juvenile stimulus animal in a social recognition test in young nNOS^−/−^ animals after 6 h and we also obtained similar findings after 24 h in old nNOS^−/−^ animals. We also found similar high investigation times of novel stimulus animals during the habituation trials in young nNOS^−/−^ animals indicating a similar heightened interest in investigating them as in wildtype control animals. Differences in findings between our study as well as that of Jüch et al. (2009) and those reported by Walton et al. (2013) may therefore have reflected the different paradigms used. In the Walton et al., study the test animal was exposed continuously to the “familiar” stimulus animal for 10 min, whereas in our study only for 4 × 1 min periods. Also, during the preference test familiar and novel animals were located in separate compartments in the Walton et al. study whereas in ours and the Jüch et al. study they were presented together to the test animal.

Our finding that the NO donor molsidomine could rescue the social recognition memory deficit in young nNOS^−/−^ mice suggests that the deficit seen in these animals may be due to insufficient NO being released from other NOS isoforms in the brain. This might therefore imply that in older nNOS animals some compensatory changes had occurred to increase NO production from other isoforms resulting in an apparent gain of function with age. Indeed, molsidomine treatment has been reported to reduce age-associated decline in passive avoidance and object recognition learning in rats (Pitsikas et al., 2005). Clearly in view of our present results it would be of interest to investigate whether molsidomine would reverse the age-related decline in olfactory learning. There are two different stages of protein synthesis upon which consolidation of memories at intermediate and long-term time points depend, with the latter starting at around 6 h post initial learning (Crow et al., 2003; Richter et al., 2005; Wanisch et al., 2008; Engelmann, 2009). A number of differences in expression of proteins involved in the regulation of mRNA trafficking, stability and translation have been reported in both the hippocampus (Kirchner et al., 2004) and olfactory bulb (Jüch et al., 2009) of young nNOS^−/−^ mice which may be associated with late stage memory consolidation impairments in these, or other proteins, which underlie improved performance in old nNOS^−/−^ animals. These different compensation possibilities clearly require further investigation.

We also found a clear age-related impairment in learning of the non-social olfactory conditioning task we used. In contrast to younger mice, wildtype control mice aged 18–24 months did not show a preference for the CS+ odor, whereas both young and old nNOS^−/−^ mice did. The age-associated impairment in this task is supported by previous studies reporting similar findings using a simpler version of the paradigm (Frick et al., 2000) and other odor-reward associations (Roman et al., 1996). As with the social recognition memory paradigm learning performance on this non-social task is likely to involve plasticity changes in both olfactory bulb and hippocampus (Bunsey and Eichenbaum, 1995; Brennan et al., 1998). Thus in two rather different olfactory memory tasks we have found clear evidence for maintained or even improved performance in old aged nNOS^−/−^ mice whereas wildtype control animals show a marked age-associated decline.

Although there is evidence for age-associated decline in olfactory sensitivity in humans, and some reports of this in rodents (Doty et al., 1984; Doty, 1989; Mobley et al., 2014), the present study did not find clear evidence for this in either wildtype of nNOS^−/−^ mice. While the old wildtype mice did show impaired learning of both social and non-social odors, they displayed normal habituation to social odors across repeated trials in the social recognition task. However, they did show longer overall investigation times of the stimulus animals which might have been as a result of compensation for impaired sensitivity, although it could equally well have been as a result of a greater motivation for social interaction compared to the old nNOS^−/−^ animals The old wildtype mice were also clearly able to locate the buried sugar during the training phase of the olfactory conditioning task, even if they could not learn to discriminate between the CS+ and CS− odors. This maintained ability to find buried food rewards in aged mice despite impaired use of odor cues to guide choice of location where to dig is in agreement with a previous study (Frick et al., 2000). Thus overall, it is unlikely that olfactory memory deficits in the wildtype animals were due to impaired olfactory perception, although we cannot rule out the possibility that this might have partially contributed.

Our findings on reversal learning in the olfactory conditioning task showed that in young animals whereas wildtype controls persevered with the original CS+ and CS− contingency after 1 day of training (4 trials) the young nNOS^−/−^ animals displayed a successful reversal. This could have been due to stronger learning of the original contingency in the wildtypes, although we found no evidence that they could learn this task more quickly than their young nNOS^−/−^ counterparts. Thus it is possible that nNOS^−/−^ mice exhibit greater behavioral flexibility in terms of adapting to new priorities. Since the old wildtype animals failed to learn to discriminate between the conditioned odors we could not test whether they had impaired reversal learning on this task, however the old nNOS^−/−^ animals failed to show a complete reversal after 1 day of trials. Although this indicated an age-associated decline in reversal learning in nNOS^−/−^ animals they showed equivalent investigation times for the conditioned odors, suggesting that they were in the process of learning the new contingency. Thus, although this would need to be confirmed using other reversal learning paradigms, it is possible that old nNOS^−/−^ animals might be at least partially protected against the normal expected age-associated deficit in this behavior. There is some evidence for up-regulation of dopaminergic (D1 receptor) signaling in nNOS^−/−^ mice (Tanda et al., 2009) and impairments in reversal learning during aging in rats are associated with altered dopaminergic signaling in orbitofrontal cortex, with behavioral deficits being reversed by local infusion of a D1 receptor agonist (Mizoguchi et al., 2010).

Although the nNOS^−/−^ mice are protected from age-associated decline in these olfactory learning tasks it is not possible at this stage to conclude that this is entirely due to reduced NO from nNOS without supporting pharmacological evidence. Other compensatory factors as a result of long-term physiological or behavioral differences and the impact of nNOS deletion throughout development could not be assessed in these experiments. For example nNOS^−/−^ mice have increased expression of the GABA transporter GAT2 and of the GABA_B_ receptor (Wultsch et al., 2007) and NO may interact with GABA_B_ to influence recognition memory (Pitsikas et al., 2003b).

We have shown previously that learning in both the social recognition memory and olfactory conditioning paradigms used in the current study are impaired by pharmacological inhibition of all NOS isoforms using L-nitroarginine (James, 2003; Sanchez-Andrade et al., 2005). Thus, as already discussed above in relation to molsidomine effects on young nNOS^−/−^ mice in the social recognition task, NO derived from other NOS isoforms might be responsible for protection against age-associated cognitive decline. There is evidence that NO derived from endothelial NOS (eNOS) plays a role in promoting both neural plasticity and learning, and combined nNOS/eNOS knockout animals show more severe hippocampal LTP deficits (O’Dell et al., 1994). The expression of eNOS, like nNOS, is also reduced during aging (Morley et al., 1996) and impaired spatial memory has been reported in late middle-aged (14–15 month old) eNOS knockout mice (Austin et al., 2013). Although there are no reports of compensatory increased expression of eNOS in young nNOS^−/−^ animals, it is possible that normal age-associated declines in eNOS might be reduced leading to preserved cognitive function in old nNOS^−/−^ animals. Further, since the nNOS knockout used in our study targets only the α variant of the gene and leaves the β and γ splice variants intact (Eliasson et al., 1997), it is possibile that these latter splice variants are contributing to NO release and preserved cognitive function in aged animals.The increased activity we observed in young nNOS^−/−^ animals using activity boxes supports previous findings using open field and elevated plus maze paradigms (Tanda et al., 2009; Walton et al., 2013). This increased activity in nNOS^−/−^ animals was primarily contributed to in the first 25–30 min after they were placed in the activity boxes and as such may have been due to an enhanced response to novelty. In agreement with a previous study (Tanda et al., 2009) we also found some evidence for increased investigation times of novel animals by young, but not old, nNOS^−/−^ animals in the social recognition task. Other studies have generally failed to show any consistent evidence for increased responses to novelty in nNOS knockouts (see Wultsch et al., 2007). Nevertheless, importantly in the context of the current experiments both old wildtype and nNOS^−/−^ animals showed significant reductions in locomotor activity indicating that that lack of nNOS expression does not protect against age-associated declines in general motor activity.

In summary we have provided the first evidence for preserved cognitive function in aged animals with deletion of nNOS (targeting the α rather than β or γ splice variants). This suggests that NO derived from nNOS may contribute to age-associated neurodegenerative changes in olfactory and hippocampal regions involved the two different olfactory memory tasks we used. There was however still an age-associated decline in reversal learning in nNOS^−/−^ animals demonstrating a reduced protection from neurodegenerative changes in frontal cortical regions. Age-related reductions in locomotor behavior were also not prevented. Further studies will be required to investigate the precise signaling pathways involved in maintenance of cognitive function in aged nNOS^−/−^ mice. Since both olfactory learning tasks used in the current study are NMDA receptor dependent (James, 2003; Sanchez-Andrade et al., 2005) it is likely that age-associated decline in performance in wildtype animals reflects impaired NMDA-NO-dependent neural plasticity, possibly as a result of reduced numbers of NMDA-receptors (Gonzales et al., 1991; Wenk et al., 1991; Pittaluga et al., 1993) or reduced efficiency of glutamate-NMDA-NO signaling triggering protein synthesis-dependent plasticity changes involved in memory consolidation (Crow et al., 2003; Richter et al., 2005; Wanisch et al., 2008; Engelmann, 2009). Thus in aged nNOS^−/−^ animals compensatory mechanisms may have somehow prevented these changes from occurring. Additionally, as discussed above, it is possible that compensatory changes might also involve altered interactions between NO and GABAergic and dopaminergic signaling.

## Conflict of Interest Statement

The authors declare that the research was conducted in the absence of any commercial or financial relationships that could be construed as a potential conflict of interest.
